# Accuracy and stability of electronic apex locator length measurements in root canals with wide apical foramen: an ex vivo study

**DOI:** 10.1038/s41405-020-00052-3

**Published:** 2020-11-17

**Authors:** Maayan Shacham, Avi Levin, Avi Shemesh, Alex Lvovsky, Joe Ben Itzhak, Michael Solomonov

**Affiliations:** Department of Endodontics, Israel Defense Forces (IDF) Medical Corps, Tel Hashomer, Derech Sheba 2, 52621 Ramat Gan, Israel

**Keywords:** Endodontic files, Apex locators

## Abstract

The aim of the current study was to determine the accuracy of electronic apex locator (EAL) measurements when using files of different sizes in roots with wide apical foramina while considering a new parameter of *stability* of EAL reading. Ten teeth with straight roots were subjected to a sequential widening of the apical foramen to 0.6, 0.7, and 0.8 mm. The roots were embedded after each enlargement stage in an alginate mold and subjected to EAL readings. Measurements were done using sequential K-file sizes and the self-adjusting file (SAF). Measurement stability was introduced as a new additional parameter. As the difference between the file size used and the apical diameter of the canal decreases, the results obtained were more accurate and stable. The stability and accuracy of the measurements coincided with each other in a statistically significant manner. Within the limitations of the present ex vivo study, it may be concluded that in straight canals with wide apical foramina of 0.6–0.8 mm, both SS K-files which fit snugly to the walls of apical foramen and the SAF file may offer both accurate and stable EAL measurements.

## Introduction

Working length (WL) determination is an essential step in endodontic treatment, and should be performed using accurate armamentarium in order to help gain a successful treatment outcome; this procedure may be performed using several methods, one of which is an electronic apex locator (EAL) measurement.^1^

There are controversies regarding the accuracy of EAL measurements concerning the file size used and diameter of the apical part and apical foramen of the root canals. Several studies suggest that accurate results can be obtained regardless of the file size used.^2–6^ Some studies support the usage of files smaller than canal diameter,^7,8^ while others claim that more accurate EAL measurements can be obtained when a file with a size close to the canal diameter is used.^9–11^ Furthermore, some studies report that apical foramen diameter above 0.6 mm leads to inaccurate EAL results.^3,4,12^

Inaccurate EAL measurement may result in either short or long measurements. If root canal procedures are performed short of the WL in vital cases, no negative consequences are to be expected.^13^ In infected cases sometimes the apical tissues are vital^14^ and short WL probably will not influence posttreatment outcomes. If necrotic, infected tissues are retained in the apical area these may lead to negative consequences.^15,16^ Long measurements are much more dangerous as it may lead to over-instrumentation and overextension of obturation.^17,18^ Direct mechanical and chemical trauma (e.g., sodium hypochlorite irrigation beyond the apex), along with massive extravasation of debris may lead to flare ups and postoperative pain,^19–22^ as well as significantly prolong the healing process^23^ or prevent healing at all.^24^ In some cases neighboring anatomical structure may also become involved and injured, including the maxillary sinus,^25^ mental^26^, and mandibular^27^ nerves.

It is commonly accepted that root canal procedures (including cleaning, shaping, and obturation) should terminate at the apical constriction of the root canal. The apical constriction is defined as the apical portion of the root canal having the narrowest diameter; position may vary but is usually 0.5–1.0 mm short of the center of the apical foramen.^28^ The apical constriction may also be missing in cases of young immature teeth in which the apical constriction has not yet fully developed, or in teeth with apical periodontitis and apical inflammatory resorption.^29^

Fully formed roots may have different geometry of apical part, e.g., a divergent apical portion of the canal wall.^17^ The apical portion of the canal walls beyond the apical constriction may be convergent, divergent, or parallel.^30–32^ In case of palatal roots of maxillary teeth, absence of apical constriction was observed in 76.6% of specimens was observed in a micro-computed tomography study.^33^ These may affect the ability to provide successful cleaning and obturation procedures. Thus it is of paramount importance to accurately determine the WL using EAL and to obtain a stable measurement. Accidental over-instrumentation may also result in a root canal with no apical constriction. In such situations, careful instrumentation and obturation should be performed in order to prevent extension beyond the wide apical foramen.^34^

The self-adjusting file (SAF) is a nickel–titanium (Ni–Ti) compressible file that adapts itself to the three-dimensional shape and size of the root canal no matter what is the canal diameter. No information is available about the influence of such adaptation on EAL measurements.

Many dental practitioners face the issue of *unstable* EAL reading, with limited literature available regarding this matter. Several authors suggested a definition for unstable EAL reading as the inability to obtain a constant reading for more than 5 s, or considered stable if the measurement remained stable for at least 5 s.^8,35^ To the best of our knowledge, no study has experimentally addressed the relationship between the *stability* of EAL and accuracy of the EAL measurement.

This ex vivo study aimed to determine the accuracy of EAL readings when using files of different increasing sizes in roots with wide apical foramina, while considering also a new parameter of *stability* of the EAL reading.

## Materials and methods

### Sampling and procedure

The study was approved by the institution’s research ethics committee (approval #18.004). The study design followed that of Herrera et al.^3^ Ten teeth were selected from a pool of recently extracted teeth that were stored in distilled water containing 10% formalin. Five teeth were single rooted teeth with a single canal. Of the remaining five teeth, three were upper molars and two were lower molars. Only the palatal and distal root canals were used in case of molar teeth. All canals were straight without any evident curves. The teeth were extracted for reasons not related to the present study. Dental X-ray images were taken from two different angles (B-L, M-D) to evaluate the root canal anatomy. The roots that were used in this study were all confirmed to have mature apices with straight canals of less than 5-degree curvature. The study included palatal roots of maxillary molars, distal roots of mandibular molars and incisors. The crowns were sectioned at the CEJ to gain standard root canal access and obtain a reproducible point of reference. ^3^ For purposes of this study, the canal length to the apical foramen (AFL) was defined as the “true length” of the canal.^3,36–39^ The AFL was determined by introducing a #10 stainless steel (SS) K-file into the canal until the tip of the file became visible at the apical foramen, using a dental operating microscope.

The canals were progressively enlarged to AFL by using SS K-files and irrigation with 2 ml of 3% sodium hypochlorite after the use of each file. The enlargement was conducted up to size #60 SS K-file (thus creating an “apical foramen” size #60). Afterward, “true length” of the canal was measured again, to verify that the apical enlargement have not changed the AFL.

### Measurements

The teeth were then embedded in an alginate mold that was kept moist with saline solution and measurements of canal length with an EAL (Apit 11, Osada, Tokyo, Japan) were performed^36^ using either SS K-files of different sizes (from #10 to #60 (KF10–KF60) or a 1.5 mm diameter SAF (Redent, Raanana, Israel). The teeth were inserted with care into the alginate mold, thus avoiding alginate entrapment within the root canal.

EAL measurements were done following the EAL manufacturer’s instructions. The canal was filled with 3% sodium hypochlorite, and the file was inserted into the canal until the reading on the apex locator dial flashed “APEX.” This mark has been reported to represent the major apical foramen.^40^ All measurements were performed during a single day by a one investigator (MS) and verified by a second investigator (AL). All measurements were performed in triplicates, and the mean value of the three measurements was taken as the result (Intraclass coefficient = 0.996).

Each root was then removed from the alginate mold, and canal enlargement was subsequently continued up to an apical foramen diameter size #70. The AFL was reestablished under the microscope to verify that it has not been changed by the enlargement procedure. The tooth was embedded again in alginate mold and length measurements with the EAL were then performed using SS K-files #10–70 and a 1.5 mm SAF file.

**Fig. 1:**
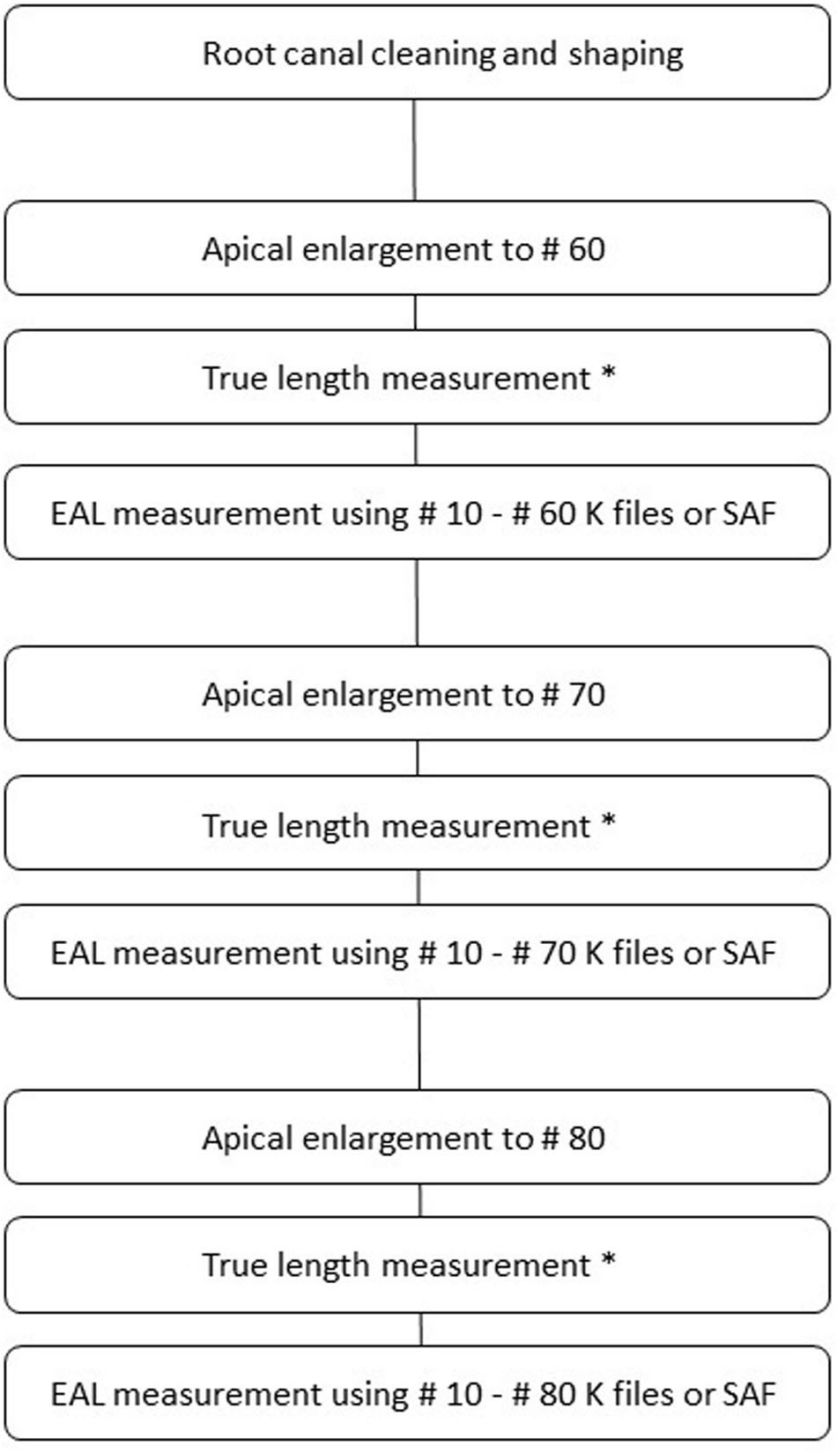
Study design. Asterisk sign indicates true length measurement using microscopic examination when the tip of the file is visible at the apical foramen. After each step, the tooth was removed from the alginate mold, the apical foramen enlarged by one size, true length was reestablished, and the tooth was embedded again in an alginate mold for the next EAL measurement.

The same process was repeated in an enlargement of the apical foramen diameter to size #80 and using SS K-files #10–80 and a 1.5 mm SAF file for canal length measurements (Fig. 1). To avoid bias, all measurements were taken while randomizing the order of the file sizes and types. All measurements were taken by the same operator.

### Stability of EAL measurement

A “stable” measurement was defined as one in which the electronic bar of the EAL steadily progressed to the “APEX” sign during file introduction through the canal to the AFL, and stayed stable when reading this point for at least 5 s. An “unstable” measurement was defined as one in which the electronic bar on the EAL display moved chaotically, irrelevant of the file movement within the canal to the AFL, or was unstable when at the “APEX” position.

### Statistical analyses

The proportions of measurements falling within tolerances of ±0.5 and ±1 mm of the “true length” were calculated for accuracy and stability measures. Statistical analysis was performed using a statistical software (SPSS 22, IBM, Armonk, NY). Data distribution was assessed using the Shapiro–Wilk test. Since no normal distribution was found, chi-square and Mann–Whitney *U* tests were used, with statistical significance set at ≤0.05.

## Results

Table 1 presents the accuracy of EAL measurements using different SS K-file sizes or the SAF in all study groups. It was found that as the difference between the size of the file used for measurements and the apical diameter of the canal decreased, the results obtained were more accurate. A difference of more than 0.2 mm between the apical diameter of the canal and the size of the SS K-file used for measurement led to inaccurate results (based on ±0.5 mm as an acceptable range of error). When using the largest SS K-file corresponding to each apical diameter in all study groups, the data showed 100% accurate EAL results. Length measurements in all three types of enlarged canals (apical foramen size #60, #70, and #80) were also performed using a 1.5 mm SAF file, which can adapt itself to canals sized #60, #70, and #80, and resulted in 100% accuracy.

**Table 1:** Accuracy of length measurement with EAL.

File used with EAL	Size of apical foramen		
	#60	#70	#80
#15	1.00 (±0.77)	1.70 (±1.71)	1.80 (±1.78)
#20	1.16 (±0.81)	1.7 (± 1.09)	1.90 (±1.55)
#25	1.00 (±0.81)	1.50 (±1.17)	2.70 (±1.3)
#30	0.44 (±0.52)	1.43 (±1.01)	1.91 (±1.39)
#35	0.50 (±0.47)	1.05 (±0.91)	1.64 (±1.49)
#40	0.45 (±0.43)	0.83 (±0.79)	0.93 (±1.08)
#45	0.25 (±0.35)	0.50 (±0.47)	0.60 (±0.93)
#50	0.30 (±0.42)	0.45 (±0.36)	0.75 (±0.97)
#55	0.15 (±0.24)	0.40 (±0.39)	0.40 (±0.65)
#60	0	0.15 (±0.33)	0.30 (±0.67)
#70	Not used	0.05 (±0.15)	0
#80	Not used	Not used	0
SAF	0	0	0

The difference between “true length” and EAL measurement is shown in mm (±SEM).

In the present study, the *stability* of an EAL reading was introduced and studied as an additional parameter of EAL function. Both the #55–80 sized SS K-files and the SAF obtained more stable results compared to the smaller-sized files. As shown in Table 2, the majority of stable results were found when the difference between the apical diameter of the canal and that of the file used for measurements was <0.5 mm. These findings indicate that more stable readings can be obtained with an EAL when the difference between the size of the file used for measurement and the apical diameter of the canal decreases. The data also indicate that the stability and accuracy of the measurements coincide with each other in a statistically significant manner (Table 2).

**Table 2:** Accuracy and stability of EAL readings.

	Difference between EAL reading and true length	
	<0.5 mm	≥0.5 mm
Unstable readings	49%^a^	51%^b^
Stable readings	95%^a^	5%^b^

Percentage of measurements with stable or unstable reading, which were done using K-file sizes #10–80 and 1.5 mm SAF file in all three enlargement groups.

Note: Values in the same row and sub-table not sharing the same superscript (i.e., a or b) are significantly different at *p* < 0.05 in the two-sided test of equality for column proportions. Tests assume equal variances.^2^ Tests are adjusted for all pairwise comparisons within a row of each innermost sub-table using the Bonferroni correction.^2^

## Discussion

The procedure used in the present ex vivo study aimed to produce an artificially enlarged apical foramen which represented clinical cases with such large apical foramina. This was different from the common clinical procedure, in which the apical constriction is preserved by defining WL as AFL −0.5 mm.^34^

The apical foramen is considered to be the end point of the root canal system, and as such it is should be precisely detected during root canal procedures.^1,41^ EAL operate by applying weak electrical current (AC) from the file’s tip to apical foramen. When closing the electrical circuit, with the absence of an insulator (i.e., dentin), an “APEX” reading will be obtained. The contact with the canal’s wall, and with it the electrolyte flux across the dentin is what makes the difference in this study. When operating in canals with wide apical foramina, the magnitude of the current (10 mA) is insufficient in order to close the electrical circuit. It’s possible to overcome this obstacle by reducing the difference between the size of the canal and the file by using a larger file.^1,3^

In the present study, the accuracy of root canal length measurements with an EAL and the stability of the EAL reading were studied in canals with wide apical foramina (sizes #60, #70, and #80). The model used here showed that in all groups both snug-fitting SS K-files and the SAF file gave 100% of accurate results, supporting previous studies, according to which the size of the file used for measurement should be in a close relation to the canal diameter.^9–11^ The SAF file can adapt itself to the canal diameter, thus fitting snugly in all three apical sizes that were studied, thereby providing accurate and stable results.

Previous studies have shown that both SS and Ni–Ti instruments may be safely used during root canal measurement with EAL.^42,43^ To the best of our knowledge, there is no data regarding the accuracy of SAF when used for EAL measurements. The adaptation of SAF to canal walls may be beneficial during clinical EAL measurements, as clinicians usually have no preliminary data regarding the true apical foramen size of a given canal. We chose to test and then present in Table 1 all file sizes, to illustrate the inadequacy of the smaller files when measurement of canal length is attempted: misleading measurements of more than 1.8 mm may be encountered when thin files (#15–30) are used for length measurement in large canals (size #80). The present model was based on previous studies, which found that when enlarging the apical foramen size to 0.6 mm, the EAL measurements are well-tolerated within an error range of ±0.5 mm,^2–6,8,9,44,45^ with the latter tolerance range considered clinically acceptable.^2,8,44,45^

Practitioners commonly encounter clinically unstable EAL readings. Venturi and Breschi and Briseño-Marroquín et al. briefly addressed this issue and described unstable reading as an inability to obtain a constant reading for more than 5 s.^8,35^ Therefore, the parameter of EAL measurement “stability” was defined in the present study as a situation in which the electronic bar of the EAL steadily progresses to the “APEX” sign during file introduction into the canal, up to the AFL, where the reading remained stable at the “Apex” position. An unstable measurement was defined as a situation in which the electronic bar display moves chaotically, irrelevant of the file movement within the canal, or is unstable at the “APEX” position.

In the present study, an intimate contact between snug-fitting SS K-files or a SAF file provided the most stable results. The close relation between the file and the canal walls is most likely responsible for the stable EAL readings. The data show that stable EAL measurements were associated with more accurate results (Table 2). This can be explained by the intimate contact between larger files and the canal walls, or by the adaptation of the SAF to the canal walls.

Several limitations of this study should be taken into consideration, as the clinical situation may be different from this ex vivo experimental model. This may be the case in (i) curved canals;^46,47^ (ii) oval canals;^48,49^ (iii) roots with a resorbed apex;^29^ and (iv) an open and divergent canal with an apical foramen diameter >0.8 mm.^50^ Further studies will be required to investigate EAL accuracy and stability in these commonly encountered situations.
